# Productive Infection of Bovine Papillomavirus Type 2 in the Placenta of Pregnant Cows Affected with Urinary Bladder Tumors

**DOI:** 10.1371/journal.pone.0033569

**Published:** 2012-03-27

**Authors:** Sante Roperto, Giuseppe Borzacchiello, Iolanda Esposito, Marita Riccardi, Chiara Urraro, Roberta Lucà, Annunziata Corteggio, Rosarita Tatè, Michele Cermola, Orlando Paciello, Franco Roperto

**Affiliations:** 1 Department of Pathology and Animal Health, Naples University Federico II, Naples, Italy; 2 Institute of Genetics and Biophysics ‘Adriano Buzzati-Traverso’, CNR, Naples, Italy; Wageningen University and Research Centre, Netherlands

## Abstract

Papillomaviruses (PVs) are believed to be highly epitheliotropic as they usually establish productive infections within stratified epithelia. *In vitro*, various PVs appear to complete their entire life-cycle in different trophoblastic cell lines. In this study, infection by and protein expression of bovine papillomavirus type 2 (BPV-2) in the uterine and chorionic epithelium of the placenta has been described in four cows suffering from naturally occurring papillomavirus-associated urothelial bladder tumors. E5 oncoprotein was detected both by Western blot analysis and immunohistochemically. It appears to be complexed and perfectly co-localized with the activated platelet-derived growth factor ß receptor (PDGFßR) by laser scanning confocal microscopy. The activated PDGFßR might be involved in organogenesis and neo-angiogenesis rather than in cell transformation during pregnancy. The major capsid protein, L1, believed to be only expressed in productive papillomavirus infection has been detected by Western blot analysis. Immunohistochemical investigations confirmed the presence of L1 protein both in the cytoplasm and nuclei of cells of the uterine and chorionic epithelium. Trophoblastic cells appear to be the major target for L1 protein expression. Finally, the early protein E2, required for viral DNA replication and known to be expressed during a productive infection, has been detected by Western blot and immunohistochemically. Electron microscopic investigations detected viral particles in nuclei of uterine and chorionic epithelium. This study shows that both active and productive infections by BPV-2 in the placenta of pregnant cows can occur *in vivo*.

## Introduction

Bovine papillomavirus type 2 (BPV-2) infection is common in cattle on pasture and grazing on bracken fern infested lands. This often results in tumors of the urinary bladder [1], [2]. Recent studies revealed BPV-2 DNA sequences in ∼50% of urinary bladder samples from free-ranging apparently healthy cattle and in ∼78% of bovine neoplastic urinary bladder samples [1], [3]. It is currently presumed that BPV-2 acts synergistically with immunosuppressive, mutagenic and carcinogenic chemicals of bracken fern in molecular pathway(s) leading to bladder carcinogenesis [1]. In more than 90% of cases, tumors of the urinary bladder cause a severe clinical syndrome known as chronic enzootic hematuria (CEH) [4].

Papillomaviruses (PVs) are believed to be highly epitheliotropic as they usually establish productive infections within stratified epithelia only [5], [6]. BPV-1/-2 are closely related serotypes and are the only ones known to infect both epithelial and mesenchymal tissues [5], [7]. Similar to other PVs, BPV-1/-2 replication and virion production appear to be confined to the epithelial region of the lesions [7]. However, new host cell types are emerging [8] and the list of new PV tissue tropisms is still increasing [9], [10], [11], [12].

Recently, a naturally occurring BPV-2 productive infection has been shown to take place in peripheral blood mononuclear cells (PBMCs) of cattle suffering from tumors of the urinary bladder [10], which demonstrates that hematogenous spreading of the virus by blood stream is an actual occurrence.

It has been reported that HPV could infect human placenta [9], [11]. However, studies evaluating the presence of PVs in intrauterine tissues including placenta are controversial in so far as data can be interpreted as evidence for an active infection or the result of contamination events [13].

It has been shown that multiple types of human papillomavirus (HPV) could infect and replicate in non-invasive trophoblast cell lines (3A trophoblast cells) [14]. Furthermore, HPV infections of the BeWo and HTR-8/SVneo cell lines have been reported [9], [15]. Both cell line systems share many phenotypic features with the extravillous, invasive trophoblastic cells of the normal human first-trimester placenta.

The aim of the present report was to document that the placenta of cows is an anatomical site of real, naturally occurring BPV-2 infection and that BPV-2 completes its life cycle in the uterine endometrial and chorionic epithelium. Therefore, this study is the first to reveal that the placenta of pregnant cows affected by BPV-2-induced urothelial bladder tumors is an additional site of BPV-2 infection, where viral proteins are expressed and virion assembly occurs.

## Materials and Methods

### Ethics Statement

In this study we did not perform any animal experiments. We collected the samples directly from public slaughterhouses; the animals were slaughtered following owner's decisions and after a mandatory clinical *ante-mortem* examination, as required by the European Union legislation.

### Immunoprecipitation of BPV-2 E5 oncoprotein in blood samples

Our veterinarian colleagues collected peripheral blood samples in tubes to perform serum investigation to eradicate brucellosis and leukosis according to Italian legislation. Four of the animals for which blood test were performed were suffering from chronic enzootic hematuria for several years as clinically determined. Blood samples of the hematuric cows were brought to the laboratory during an ongoing study on papillomavirus biology [10]. The peripheral blood mononuclear cell samples proved positive for BPV-2 E5 oncoprotein [10].

### Tissue Samples

The hematuric cows were slaughtered at public slaughterhouses in southern Italy; they were found to be pregnant. Post-mortem examination revealed the presence of severe neoplastic lesions scattered on the bladder mucosa. Bladder and placenta tissue specimens were sampled. To prevent possible cross-contaminations, each sample was immediately divided into several parts that were frozen in liquid nitrogen for subsequent molecular biological analysis, or fixed in 10% buffered formalin for protein expression assays.

### Histopathology

Tissues fixed in 10% neutral buffered formalin were routinely processed for paraffin embedding. Histologic diagnosis of bladder tumors was assessed on 5-µm-thick hematoxylin-eosin (HE)-stained sections following recently suggested morphological criteria [2].

### BPV-2 DNA Detection

DNA was extracted from placental samples from cows affected with urothelial bladder tumors as well as from apparently healthy cows using the DNeasy Tissue Kit (Qiagen) according to the manufacturer's protocol. All the samples were lysed using proteinase K. Lysates were loaded onto DNeasy spin columns. After two washings pure DNA was eluted in low salt buffer. For the detection of BPV-2 DNA specific primers for the E5 region were designed by Beacon Designer 2.0 software as reported elsewhere [16]. The forward primer BPV-2NS (5′-TACTGTTTCTGCTGCTATTT-3′) and the reverse primer BPV-2NAS (5′-ACAAATCAAATCCACATAATAGTA-3′) amplified a fragment of 125 bp from 3943 to 4067 of complete genome of BPV-2. To evaluate the adequacy of the DNA, a control PCR for bovine β-actin sequence was performed using a set of primers (forward, 5′-GAGCGTGGCTACAGCTTCAC-3′; reverse, 5′-CATTGCCGATGGTGATGA-3′).

Aliquots 50–100 ng of purified DNA were amplified in 25 µl of reaction mixture containing 1.5 mM MgCl_2_ for β-actin primers and 2 mM for BPV-2 primers, 200 mM each dNTP, 480 nM of each primer and 2.5 U of AmpliTaq Gold DNA Polymerase (Applied Biosystems, Monza, Italy). The reaction was carried out in a thermocycler (Veriti, Applied Biosystems) with an initial denaturation step of 2 min and 30 sec. Then, 35 cycles of amplification were carried out with a denaturation step at 95°C for 30 sec, an annealing step at 60°C, 1 min, for β-actin or at 50°C, 30 sec, for BPV-2, and an extension step at 72°C for 1 min. A final extension step at 72°C for 5 min was performed in each PCR assay. Detection of the amplified products was carried out by electrophoresis on ethidium bromide-stained agarose gel. In each experiment, a blank sample consisting of reaction mixture without DNA and a positive sample consisting of BPV-2 clone DNA (a kind gift by Dr. A. Venuti) were included. Five µl of the amplified products were subjected to a second run of PCR under the same experimental conditions. Amplified products from the last PCR were electrophoresed in a 2,5% agarose gel and visualized by ethidium bromide stain. The quality of DNA was tested with primers for bovine β-actin gene.

### BPV-2 E5 Immunoprecipitation

Tissue samples from placenta were lysed in ice-cold buffer containing 50 mM Tris-HCl (pH 7.5), 1% (v/v) Triton X-100, 150 mM NaCl, 2 mM PMSF, 1.7 mg/ml Aprotinin, 50 mM NaF, and 1 mM sodium orthovanadate. The protein concentration was measured using the Bradford assay (Bio-Rad Laboratories, Milan, Italy). Proteins derived from bladder and placenta (1000 µg) were immunoprecipitated by using 2 µg of a polyclonal sheep anti-E5 antibody (a kind gift by Dr. M.S. Campo) and 30 µl of Protein A/G-Plus Agarose (Santa Cruz Biotechnology, CA, USA). Immunoprecipitates were washed four times in complete lysis buffer (above), finally heated in 1X Laemmli sample buffer at 100°C for 10 min. Immunoprecipitates were separated on polyacrylamide gels and transferred to nitrocellulose filter membranes (Ge Healthcare Life Sciences, Chalfont St Giles, UK) for 16 h at 30 mA in 192 mM glycine/25 mM Tris-HCl (pH 7.5)/10% methanol. Membranes were blocked for 1 h at room temperature in 5% non-fat dry milk, incubated with anti-E5, anti-PDGFβR and anti-Tyr^770^ phosphorylated PDGFβR antibodies (Santa Cruz Biotechnology, CA, USA) overnight at 4°C. After three washes in Tris-buffered saline, membranes were incubated with rabbit anti-sheep IgG-horseradish peroxidase (HRP) (Santa Cruz Biotechnology, CA, USA) or with goat anti-rabbit IgG-HRP (Bio-Rad Laboratories, Milan, Italy) for 60 min at room temperature. Proteins were visualized by enhanced chemiluminescence system (Western Blotting Luminol Reagent, Santa Cruz Biotechnology, CA, USA).

### Immunohistochemistry

Placental sections from both cows affected with urinary bladder tumors and from apparently healthy cows were processed with the same procedures. Briefly, the sections were deparaffinized and then endogenous peroxidase activity was blocked by incubation in 0.3% H_2_O_2_ in methanol for 20 min. Antigen retrieval was performed by pretreating with microwave heating (twice for 5 min each at 750 W) in citrate buffer pH 6.0. The slides were washed three times with phosphate buffered saline (PBS), pH 7.4, 0.01 M, then incubated for 1 h at room temperature with rabbit serum (Sigma-Aldrich, Milan, Italy) diluted at 1 in 10 in PBS. The excess serum was drained off and a polyclonal sheep anti-BPV-2 E5 primary antibody (a kind gift by Dr. M.S. Campo) diluted at 1 in 40,000 in PBS, was applied for 1 h at room temperature in a humid chamber. Following incubation, the sections were rinsed three times for 5 min with PBS before application of the rabbit anti-sheep biotinylated secondary antibody (Santa Cruz Biotechnology, Inc., CA, USA), diluted at 1 in 100 in PBS for 45 min at room temperature. For E2 and L1 detection the slides were washed three times with PBS, pH 7.4, 0.01 M, then incubated for 1 h at room temperature with protein block serum-free (DakoCytomation, Denmark). Polyclonal rabbit anti-BPV-2 E2 primary antibody (a kind gift by Dr. E. Androphy) diluted at 1 in 200/300 in PBS and a monoclonal mouse anti-HPV-16 L1 (late protein) (Chemicon International, CA, USA) diluted at 1 in 200 in PBS were applied overnight at 4°C in a humid chamber. The sections were rinsed three times for 5 min with PBS, incubated for 40 min at room temperature with appropriate biotinylated secondary antibody (labelled streptavidin-biotin (LSAB) Kit; DakoCytomation, Denmark). Finally, all the sections were washed three times with PBS and then incubated with streptavidin-conjugated to horseradish peroxidase (LSAB Kit; DakoCytomation, Denmark). Color development was obtained by treatment with diaminobenzidine (DakoCytomation, Denmark) for 5–20 min. Sections were counter stained with Mayer's hematoxylin.

### Immunofluorescence

Two-color immunofluorescence staining was performed with all placental samples to assess the presence of E5 and PDGFβR also in uterine and chorionic epithelium. Briefly, the sections were deparaffinized, rinsed in PBS and heated in a microwave oven in citrate buffer (as above) to allow antigen unmasking. Slides were then pre-incubated with normal donkey serum diluted at 1 in 20 in PBS for 30 min, and overlaid with polyclonal sheep anti-E5 diluted at 1 in 50 in PBS for 2 h at room temperature in a humid chamber. Then, a polyclonal goat anti-p-PDGFβR antibody (Santa Cruz Biotechnology Inc., CA., U.S.A.) was applied overnight diluted at 1 in 50 in PBS. A secondary antibody Alexa Fluor 488 donkey anti-sheep (Invitrogen, Molecular Probes) and a secondary antibody Alexa Fluor 546 donkey anti-goat (Invitrogen, Molecular Probes), diluted at 1 in 100 in PBS, were applied for 2 h at room temperature.

After washing 3 times with PBS, the slides were mounted under aqueous medium (Sigma-Aldrich, Milan, Italy). For observation and photography, a laser scanning confocal microscope LSM-510 (Zeiss, Göttingen, Germany) was used.

### Western blot analysis for total and phosphorylated PDGFβR, E2, L1

Placental samples were solubilized at 4°C in lysis buffer containing 50 mM Tris-HCl pH 7.5, 150 mM NaCl, 1% Triton X-100. Immediately prior to use, the following reagents were added: 1 mM DTT, 2 mM PMSF, 1.7 mg/ml Aprotinin, 25 mM NaF, 1 mM Na_3_VO_4_ (Sigma-Aldrich, Milan, Italy).

Lysates were clarified at 21,500× *g* for 30 min. The protein concentration was measured using the Bradford assay (Bio-Rad Laboratories, Milan, Italy). For Western blotting, 50 µg of lysate proteins were heated at 100°C in 4X premixed Laemmli sample buffer. Proteins were subjected to sodium dodecyl sulfate–polyacrylamide gel electrophoresis (SDS–PAGE) (7.5% polyacrylamide) under reducing conditions.

After electrophoresis, proteins were transferred onto nitrocellulose filter membranes (GE Healtcare Life Sciences, Chalfont St Giles, UK) for 1 h at 350 mA in 192 mM glycine/25 mM Tris-HCl (pH 7.5)/10% methanol. The membranes were blocked with 5% non-fat dry milk in Tris-buffered saline (TBS, pH 7.5) for 1 h at room temperature, washed with TBS-0.1% Tween. Then, membranes were probed with Tyr^770^-phosphorylated anti-PDGFβR and non-phosphorylated anti-PDGFβR antibodies (Santa Cruz Biotechnology, CA, USA), an anti-E2 antibody and anti-L1 antibody (clone BPV1-1H8, AbCam, Cambridge, UK) for an overnight incubation at 4°C. After three washes in Tris-buffered saline, membranes were incubated with horseradish peroxidase-conjugated anti-rabbit IgG (Santa Cruz Biotechnology, CA, USA) or anti-mouse IgG (Bio-Rad Laboratories, Milan, Italy) respectively, for 1 h at room temperature. After appropriate washing steps, bound antibody was visualized by an enhanced chemiluminescence system (Western Blotting Luminol Reagent, Santa Cruz Biotechnology, CA, USA).

### Transmission Electron Microscopy

Formalin-fixed placenta was cut into small pieces. They were washed in 0.1 M phosphate buffer (pH 7.4) for 20 min 5 times and post fixed in 1% OsO_4_ in phosphate buffer (pH 7.4) for 1 h. They were washed in 0.1 M phosphate buffer (pH 7.4), and then dehydrated through graded alcohols, and embedded in Epon 812 epoxy resin (Polyscience, Niles, IL, USA). The whole process of inclusion was performed using the Leica EM TP automated routine tissue processor station (Leica Microsystems). Ultra thin sections (60–70 nm) were cut on an EM UC6 ultramicrotome (Leica Microsystems) and collected onto 300-mesh Formvar-coated grids. Sections were counterstained with uranyl acetate and lead citrate and examined with a JEOL JEM-1011 transmission electron microscope (JEOL, Tokyo, Japan) equipped with a thermionic tungsten filament and operated at an acceleration voltage of 100 kV. Images were taken using a Morada cooled slow-scan CCD camera (3783X2672 pixels) and micrographs were taken with iTEM software (Olympus Soft Imaging System GmbH, Munster, Germany). The same procedure was used to obtain ultra thin sections from placenta of unaffected, healthy cows.

## Results

Histological examination of the bladder tumors detected microscopic patterns consistent with low grade papillary carcinomas (three cases) and with high grade papillary carcinoma (one case) and all of them were positive for E5 oncoprotein as reported elsewhere [2].

**Figure 1:**
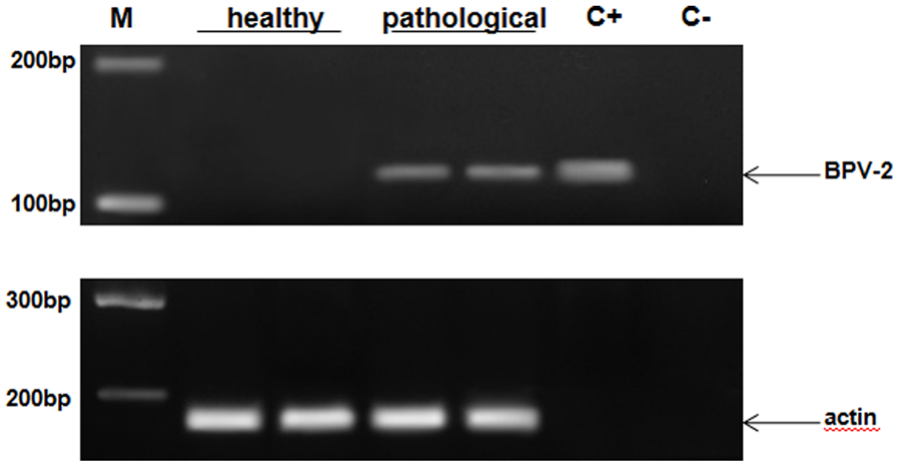
PCR amplification of DNA samples. Lane M, molecular mass marker (HyperLadder II Bioline); lanes 1–2 placental samples from healthy animals without BPV-2 DNA; lanes 3–4 placental samples from two of four animals affected with urinary bladder tumors showing BPV-2 DNA; lane C+, positive control; lane C−, negative control. The arrow indicates the position of the 125 bp BPV-2 PCR product.

**Figure 2:**
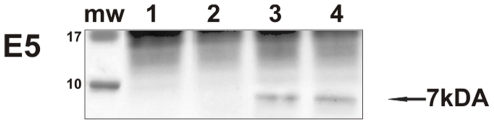
E5 immunoprecipitation. The presence of E5 protein detected by immunoprecipitation. Lanes 1–2: placenta from healthy cows. Lanes 3–4: placenta from two of four cows with papillomavirus-associated tumors of the urinary bladder.

**Figure 3:**
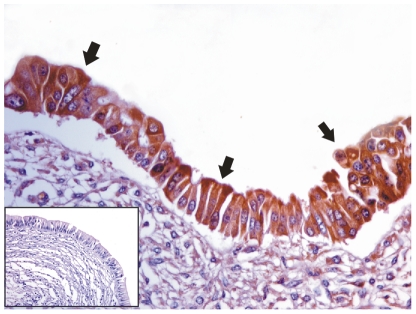
E5 immunohistochemistry. The presence of E5 oncoprotein seen by immunohistochemistry in the cells of chorionic epithelium (black arrows). Insert: corresponding negative control section.

DNA of PCR-quality was recovered from all the placental samples. PCR analysis demonstrated the presence of BPV-2 DNA only in the samples from cows affected with urothelial bladder tumors in which a fragment of the expected size (125 bp) was amplified (Figure 1). In these placental samples, the E5 oncoprotein was also detected by immunoprecipitation (Figure 2) and both in the uterine endometrial and chorionic epithelium by immunohistochemistry. E5 was found to be located mostly in the cytoplasm; however, it was also seen associated to the membrane (Figure 3).

**Figure 4:**
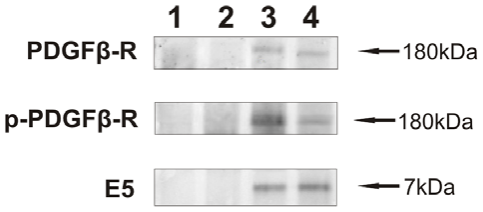
E5-PDGFß-R co-precipitation. The presence of total and phosphorylated PDGFß-R was detected in E5 immunoprecipitates. Lanes 1–2: placenta from healthy cows. Lanes 3–4: placenta from two of four cows with papillomavirus-associated tumors of the urinary bladder.

**Figure 5:**
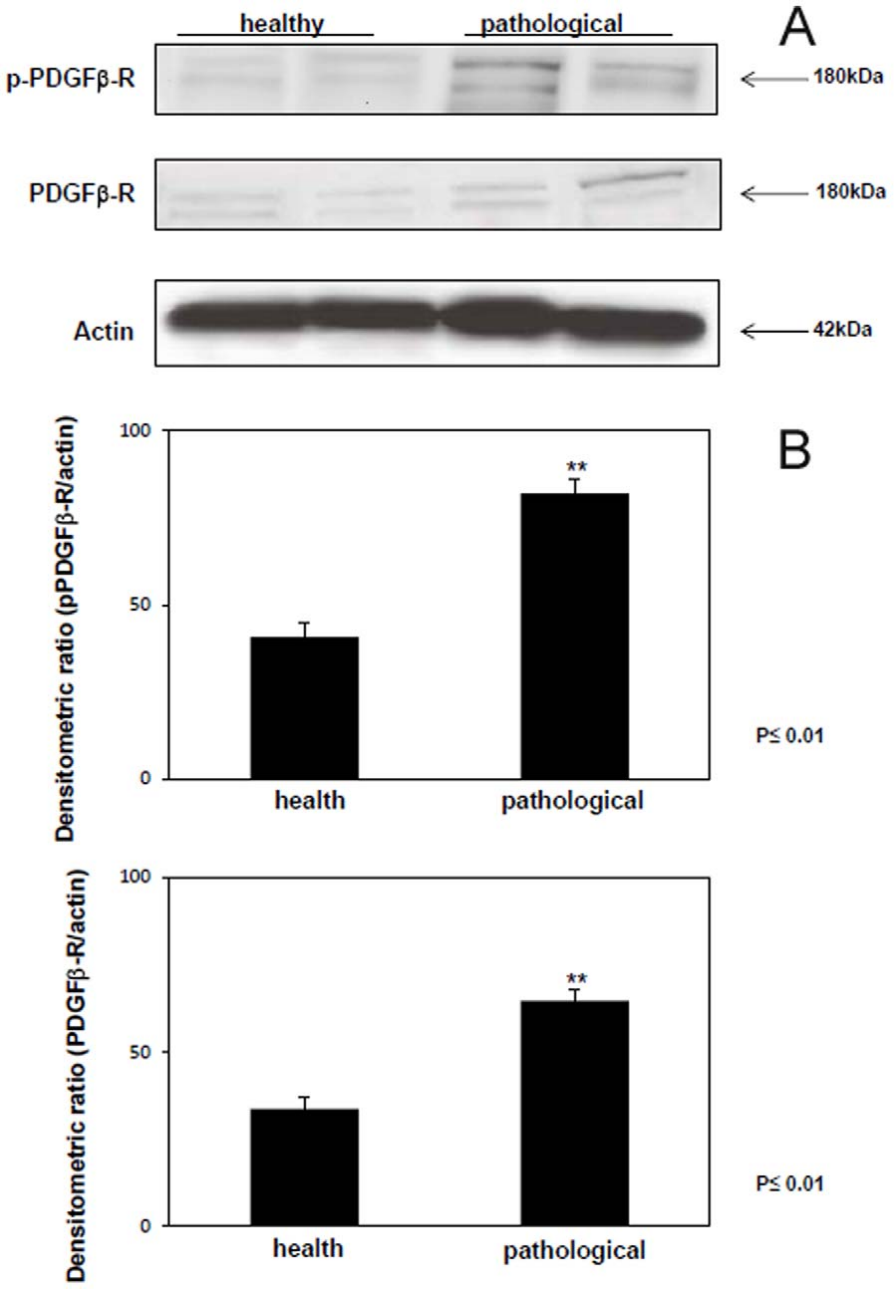
PDGFβ-R overexpression and p-PDGFβ-R activation. (A) Total protein extracts from placental tissue lysates were generated and used in Western blot analysis with an antibody specific for total PDGFβ-R and a phosphospecific PDGFβ-R antibody that recognized p-PDGFβ-R phosphorylated at Tyr770. Lanes 1–2: healthy animals. Lanes 3–4: placental tissue from two of the four cows with papillomavirus-associated tumors of the urinary bladder. Actin protein levels were detected to ensure equal protein loading. (B) Quantitative densitometric analysis of the gels was performed with Image Lab software (ChemiDoc; Bio-Rad Laboratories) and significance determined by the Student T-test (P≤0.01).

**Figure 6:**
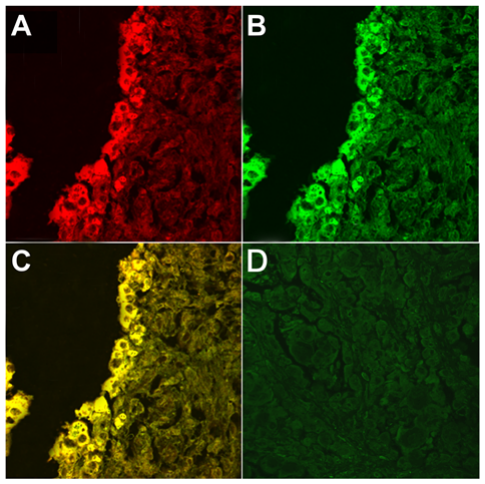
E5-pPDGFβ-R co-localization. E5-pPDGFβ-R co-localization shown in chorionic epithelium by laser scanning confocal microscopy. (A) E5 expression in red. (B) pPDGFβ-R activation in green. (C) Co-localization of the proteins in yellow. (D) Epithelium of unaffected placenta from a healthy cow showing no immunofluorescence for E5.

Western blot analysis from E5 immunoprecipitates revealed the presence of total and phosphorylated (activated) PDGFβR, indicating the two proteins were in a physical complex (Figure 4). The receptor protein appeared to be overexpressed and its phosphorylation was increased in the placental samples from cows suffering from urinary bladder tumors compared to the healthy ones as detected in total lysates (Figure 5). Morphologically, PDGFβR expression appeared to occur in the epithelium lining villi and criptae of placentomes and in the epithelial cells of interplacentomal regions. The activated PDGFβR was complexed and co-localized both at membrane and cytoplamatic levels with the E5 oncoprotein as documented by laser scanning confocal microscope (Figure 6).

**Figure 7:**
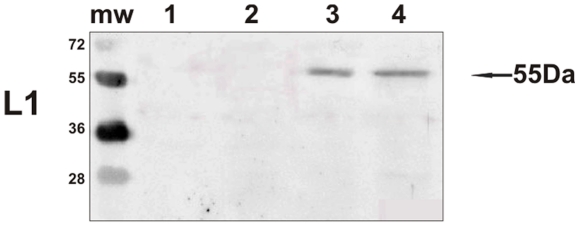
L1 detection. The presence of L1 protein detected in the placenta by Western blot. Lanes 1–2: placenta from healthy cows. Lanes 3–4: placenta from two of the four cows with papillomavirus-associated tumors of the urinary bladder.

**Figure 8:**
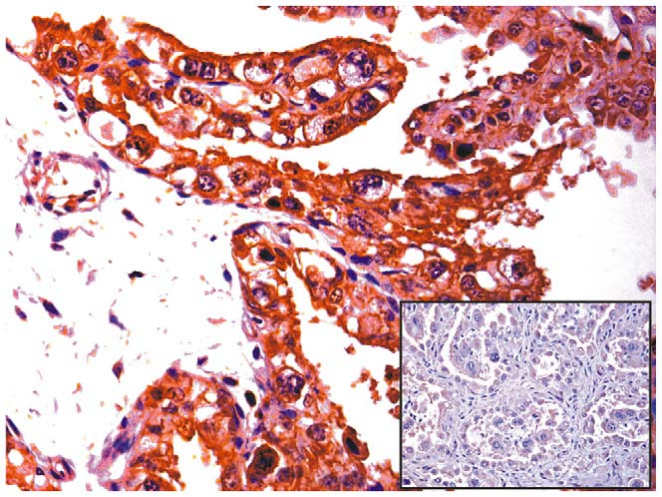
L1 immunohistochemistry. Immunohistochemical analysis confirmed the presence of L1 protein, the major viral structural protein, in the uterine and chorionic epithelium. Insert: corresponding negative control section.

As it is still controversial whether productive infections can take place naturally in the placenta, we investigated the expression of the major capsid protein, L1, believed to be only expressed in productive infections of papillomavirus [5], [17]. Western blot analysis revealed L1 expression in total protein extracts from placental samples of cows affected with urinary bladder tumors only (Figure 7). Immunohistochemical investigations confirmed the presence of L1 protein in the cytoplasm and nuclei of cells of the uterine endometrial and chorionic epithelium. Trophoblastic cells appeared to be the major target for L1 protein expression (Figure 8).

**Figure 9:**
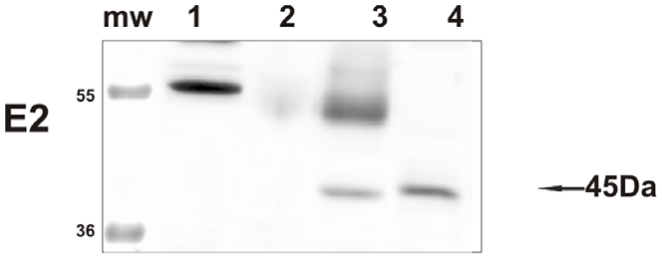
E2 detection. The presence of E2 protein detected in the placenta by Western blot. Lanes 1–2: placenta from healthy cows. Lanes 3–4: placenta from two of the four cows with papillomavirus-associated tumors of the urinary bladder.

**Figure 10:**
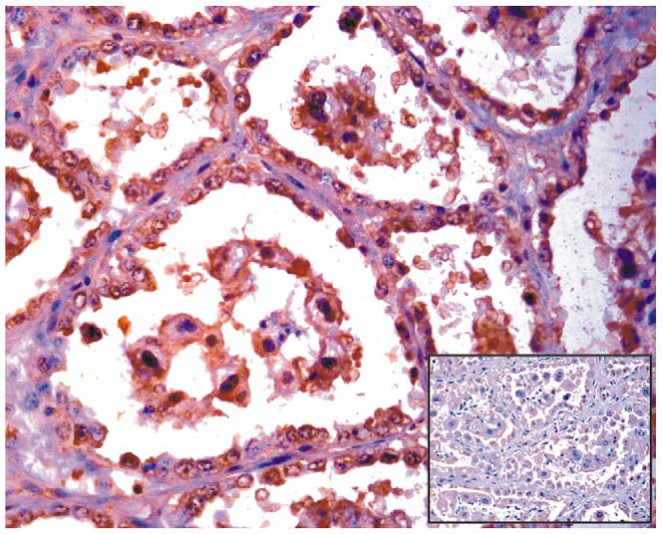
E2 immunohistochemistry. Immunohistochemical detection of E2 protein in the uterine and chorionic epithelium. Notice the nuclear immunoreactivity. Insert: corresponding negative control section.

**Figure 11:**
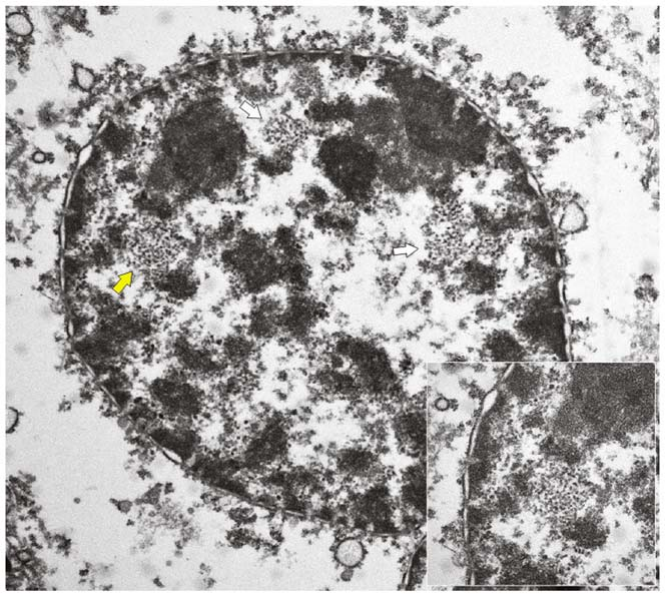
Presence of viral particles. Electron microscopical examination of a nucleus of trophoblastic cell showing randomly scattered electron dense viral particles (white and yellow arrows). ×15,000. Insert: higher magnification of viral particles shown by yellow arrow. ×50,000.

**Figure 12:**
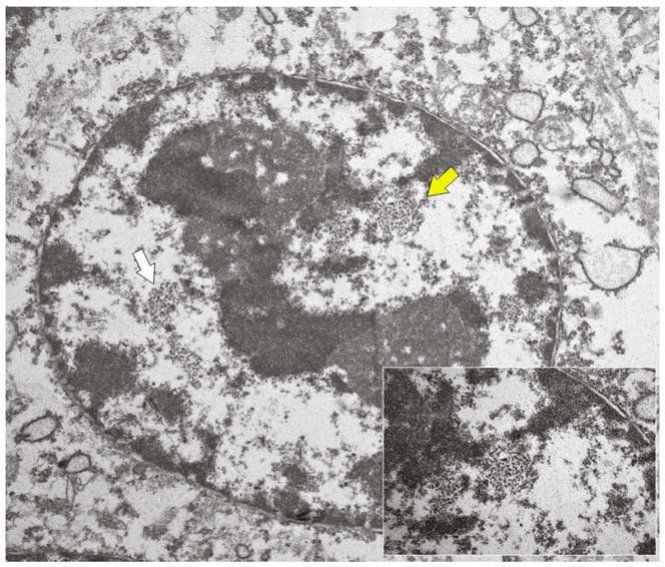
Presence of viral particles. Electron microscopical examination showed numerous clusters of chromatin-associated electron dense particles, 45–50 nm in diameter identified as viral particles (yellow and white arrows) in the nucleus of a trophoblastic cell. ×15,000. Insert: higher magnification of viral particles shown by yellow arrow. ×50,000.

**Figure 13:**
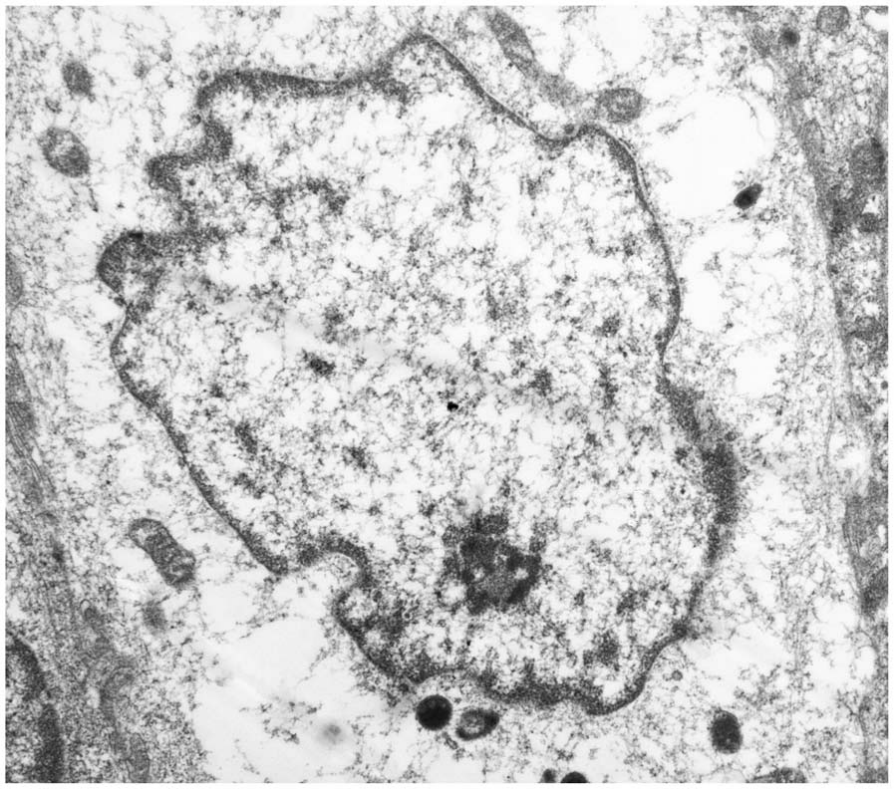
Placentomal epithelium. Nucleus of a trophoblastic cell from an uninfected placenta of a healthy cow without any electron dense particles. ×15,000.

As it has been suggested that the early protein E2, involved in a number of processes that are essential for the viral life-cycle, is among the first proteins to be expressed during a productive infection [16], we studied the expression of this protein. E2 was detected by Western blot and its expression was confirmed immunohistochemically. It was seen both in the cytoplasm and, mostly, in nuclei of epithelial cells of placentome and of interplacentomal regions of cows affected with urinary bladder tumors only (Figures 9 and 10). Ultrastructurally, electron dense particles, 45–50 nm in diameter and identified as viral particles, were seen in uterine and chorionic epithelium. They were manifested as chromatin-associated clusters or randomly scattered in the nuclei (Figures 11 and 12). No electron dense particles were seen in nuclei from placenta of unaffected, healthy cows (Figure 13).

## Discussion

Our study provides evidence for a productive BPV-2 infection in the placenta of cows suffering from naturally acquired, BPV-2-induced bladder tumors. Microscopic and submicroscopic investigations show that, in analogy to HPV infection in man [18], bovine placental trophoblasts are also the target for BPV infections. Unfortunately, ultrastructural studies were performed on formalin-fixed tissues, which have not allowed us to follow routine procedures to obtain an accurate preservation of cell structures. It is worthwhile noting that various HPV types are able to complete their life-cycle in cultured trophoblasts [14], [15], [19]. In addition, HPV DNA has been found in placental trophoblastic cells from women with cervical and/or oral HPV infection history [13] and in trophoblasts of pathological placentas from women with spontaneous abortion [18]. Recently, HPV L1 DNA has been detected in the placenta of women suffering from reproductive disorders and of a patient affected by epidermodysplasia verruciformis (EV), a skin disease characterized by an abnormal genetic susceptibility to a group of HPVs [15], [20]. Furthermore, HPV-16 and HPV-62 E6/E7 DNA was detected in the transabdominally obtained placental cells from apparently healthy, pregnant women [11]. DNA sequences of BPV-1, BPV-2 and BPV-4 were also detected in the placenta and amniotic fluid of pregnant cows although clinical implications of these results remain to be elucidated [21]. It has been suggested that gene expression of HPVs in trophoblastic cells is regulated by pregnancy hormones such as endogenous progesterone [22]. Current findings support the view that PVs are not strictly keratinocyte-specific. We have recently shown that blood cells, particularly CD4+ and CD8+ lymphocytes of hematuric cattle, are additional sites of a productive BPV-2 infection [10]. It has also been shown that experimental infection of foals by BPV-1 comprises a viraemic phase [23]. Therefore, we believe that placental BPV-2 infection may preferably take place via bloodstream although alternative routes also exist. A similar pathway has been hypothesized to occur in man [24], [25]. However, the viraemic phase has not been confirmed for HPV and hematogenous transmission of HPV remains a controversial issue [26]. Bodaghi et al. detected HPV-16 DNA in the blood of sexually naïve children that had acquired HIV-1 infection via blood transfusion [27]. Furthermore, our data emphasizes that BPV-2 E5 oncoprotein binds to the activated PDGFßR in the placental trophoblasts, which raises a number of important questions that warrant further investigations.

Genetic and biochemical data indicate that the activation of the PDGFβR is an important molecular pathway by which E5 oncoprotein becomes responsible for cell transformation in a variety of cell systems [28]. E5 forms a stable complex with the PDGFβR and induces dimerization of the receptor, activation of the tyrosine kinase activity, autophosphorylation of the intracellular domain of the receptor on tyrosine, mitogenic signaling and cell transformation [29]. However, it has also been shown that E5, when retained in the Golgi, still induces PDGF receptor autophosphorylation but does not transform cells [30].

What is the biological significance of the E5-pPDGFβR complex in the placenta? Tumors of the placental epithelium have not been clearly substantiated in domestic animals [31]. Congenital tumors associated with papillomavirus infection are extremely rare in cattle [32], [33], [34], suggesting that E5 in the placenta could rarely be responsible for cell transformation. It is conceivable that the pregnancy period might not be enough for cell transformation by E5/PDGFβR complex. It has been suggested that successful signaling through PDGFβR requires that E5 be engaged to the receptor for a sufficient period of time to induce downstream signaling [30]. Accordingly, the activation of the PDGFβR has been shown to play an important role in *in vivo* bladder carcinogenesis only in adult cattle as tumors of the urinary bladder are known to occur in 4–8-year-old cattle [2], [35].

Alternatively, the activation of the PDGFβR by E5 oncoprotein during *in utero* infections could play an important role also in non-carcinogenetic events.

Trophoblasts share many properties with malignant cancer cells and are rich in receptors for many growth factors including the PDGF receptor [36]. PDGFβR signaling is required for the maturation of the trophoblasts and fetal placental vasculature as well [37], [38], thus playing a crucial role in mammalian development [37]. The PDGFβR expression is dynamic being low *in vivo* during development but increasing dramatically during inflammation [39]. It is well-known that PDGFβR is expressed in mesenchyme, particularly in vascular smooth muscle cells (SMCs) and pericytes. It is involved in the angiogenesis and organogenesis of developing kidneys and lungs [37]. More recently, PDGFβR signaling has been shown to be important also in cardiac development [40].

In our study, the upregulation of PDGFβR prompts the intriguing hypothesis that PDGFβR signaling can be involved in an abnormal organogenesis that might lead to a compromised gestation. We found this complex also in fetal organs (Roperto et al., manuscript in preparation). Therefore, placental infections with BPVs may ultimately be associated with placental defects and abnormal fetal development resulting in reproductive disorders. It has been suggested that HPV infection can be responsible for a severe placental dysfunction resulting in preterm delivery and spontaneous abortion [15], [22], [41]. Prenatal transmission is a known mode of vertical papillomavirus transmission and an actual mechanism of virus infection of the fetus via transplacental transmission appears to take place both in humans [13], [20], [26], [42], [43], [44] and in cows [21], [45].

According to Castellsagué et al. [46], future studies are needed to establish sensitive detection methods for reliable distinction between latent and active/productive HPV. This may e.g. be achieved by the use of qPCR-based assays with probes designed to specifically recognize viral mRNA splicing products.

To date, BPV infection has not been thought important in placental abnormalities in cows. Our findings provide a scientific basis for further investigations about the role of BPV in pregnant cows in compromising placental function that may result in adverse pregnancy outcomes. It is worthwhile noting that the incidence of reproductive disorders, like infertility and abortions, caused by infectious agents is continuously increasing thus leading to substantial economic losses [47]. This is even more so as the major causes of miscarriage are only rarely identified [8].
